# Nutrient Utilization and Gut Microbiota Composition in Giant Pandas of Different Age Groups

**DOI:** 10.3390/ani14162324

**Published:** 2024-08-12

**Authors:** Chengdong Wang, Wenwen Deng, Zhi Huang, Caiwu Li, Rongping Wei, Yan Zhu, Kai Wu, Chengyao Li, Linhua Deng, Ming Wei, Xuemei Chen, Desheng Li

**Affiliations:** China Conservation and Research Centre for the Giant Panda, Key Laboratory of SFGA on the Giant Panda, Chengdu 610051, China19983169079@163.com (Z.H.);

**Keywords:** giant panda, age, feed intake, nutrient apparent digestibility, gut microbiota

## Abstract

**Simple Summary:**

The dietary structure and gut microbiota of giant pandas play an important role in nutrient utilization. The relationship between age-related dietary changes, nutrient digestibility, and gut microbiota composition is not fully understood. According to this study, bamboo consumption dramatically decreases with age. Meanwhile, a notably higher amount of bamboo shoots are consumed by geriatric giant pandas (GGP), which may be an important source of nutrients for them as bamboo shoots have a strong positive correlation with the concentration of crude protein (CP) and specific amino acids. In addition, the digestibility of crude fiber (CF) and certain amino acids are significantly correlated with the abundance of specific bacterial genera, highlighting that an age-related shift in gut microbiota has an influence on nutrient utilization. Our study provides new insights into the further study on the mechanisms underlying the effects of certain gut microbiota on nutrient digestion.

**Abstract:**

Proper feeding and nutrition are vital for maintaining the health of giant pandas (GPs), yet the impact of dietary changes and gut microbiota on their nutrient utilization remains unclear. To address these uncertainties, we investigated nutrient intake and apparent digestibility, as well as gut microbiota composition across different age groups of giant pandas: sub-adults (SGPs), adults (AGPs), and geriatrics (GGPs). Our findings revealed notable shifts in dietary patterns from SGPs to GGPs. As they aged, significantly more bamboo shoots and less bamboo were consumed. Consequently, GGPs showed significantly reduced crude fiber (CF) intake and digestibility, while crude protein (CP) did not alter significantly. In addition, 16S rRNA microbial sequencing results showed that *unidentified_Enterobacteriaceae* and *Streptococcus* were the dominant genera among all age groups. The relative abundance of the genus *Enterococcus* in GGPs was significantly higher than that in SGPs and AGPs (*p* < 0.05). Overall, our results indicated the importance of bamboo shoots as a major source of protein in GGPs’ diet, which can effectively compensate for the certain nutritional loss caused by the reduction in bamboo intake. Age-related changes in bacterial abundance have an effect on specific nutrient apparent digestibility in the gut of GPs. The data presented in this study serve as a useful reference for nutritional management in different ages of GPs under healthy conditions.

## 1. Introduction

Despite being a member of the Carnivora order, the giant panda (*Ailuropoda melanoleuca*, GP) has evolved into an herbivorous animal that mainly consumes subalpine bamboos over a long period of time [1,2]. In the wild, large quantities of bamboo have to be consumed to keep GPs from starvation and malnourishment as it is a high-fiber food with low nutritional content [1,3]. For healthy captive GPs, in addition to bamboo and bamboo shoots as their main diet, an appropriate amount of concentrated feed (mainly composed of soya beans, maize, rice, vegetable oil, egg vitamin complex, salt, and calcium and phosphorus additives), apples, and carrots, etc. are also added in their daily diet to satisfy their nutritional needs.

Nutrients provide animals with raw materials that support internal metabolism and facilitate and regulate a large array of cellular processes [4]. The macronutrients, including carbohydrates, proteins, and fats, are required in large amounts to maintain energy balance and support the physiologic needs of animals [5]. Aside from that, other nutrients which animals cannot synthesize or synthesize inadequately to meet their physiological needs, such as essential amino acids, must come from their external diet [6]. The nutritional status of GPs is fundamental to all aspects of health, including growth, reproduction, and disease resistance. Therefore, appropriate feeding and nutrition are crucial to a GP’s preventive medical management.

Nutrient digestibility is a crucial metric used in the evaluation of diet formulations, as it is affected by the state of individual GPs and their food components. It is known that with aging, physiological and metabolic changes occur [7,8]. Aging-related functional changes to the gastrointestinal system, such as altered enzyme activity and poorer circulation, may lead to a reduction in food digestion and absorption [9]. Remarkably, in comparison to sub-adult giant pandas (SGPs) and adult giant pandas (AGPs), the capacity to chew and digest bamboo, especially bamboo stalks, is reduced in geriatric giant pandas (GGPs). Consequently, in order to compensate for the inadequate nutritional intake brought on by the restriction of bamboo intake, geriatric GPs would receive an increased amount of bamboo shoots and concentrated feed according to their physical condition and preferred diets. However, the effects of feed changes on nutrient intake and digestion are unclear.

The gut microbiota, which possesses a wide variety of enzymes that break down and absorb incoming dietary nutrients and regulate nutrient metabolism conducted by the host, plays an important role in digestion [10,11]. It is well known that there exists a complex interplay between the gut microbiota of the GP and its dietary intake [12,13,14]. For example, the gut bacteria, such as *Streptococcus* spp., *Pseudomonas* spp. and *Enterococcus* spp. could benefit lignocellulose digestion [15], while other studies have confirmed that different bamboo parts and GPs’ bamboo species preference play a critical role in nutrient utilization and gut microbiota composition [3,16]. In general, the gut microbiota undergoes extensive changes across the lifespan [17]. Moreover, age-related changes in the gut microbiota of GPs have been observed in previous studies [18,19]. However, information regarding the correlations between nutrient digestibility and the gut microbiome in GPs is limited.

Therefore, the aim of this study was to elucidate the changes in nutrient intake, apparent digestibility, and gut microbiota in GPs across different age groups, especially the differences in GGPs compared with the SGPs and AGPs. In addition, age-related changes in the gut microbiota and their relationships with nutrient apparent digestibility were explored.

## 2. Materials and Methods

### 2.1. Sample Collection

**Figure 1:**
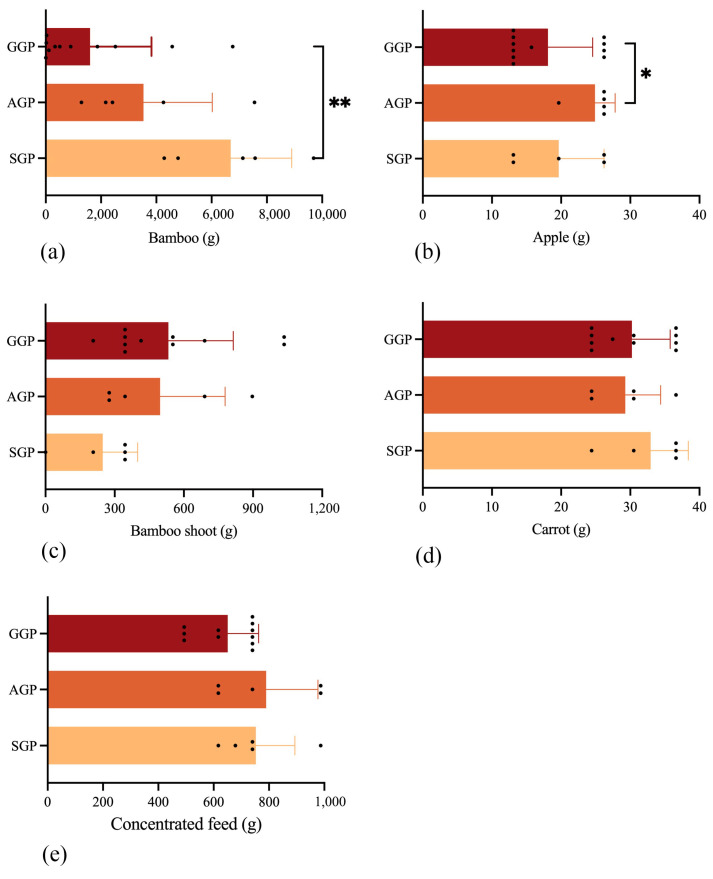
Daily intake of (**a**) bamboo, (**b**) apple, (**c**) bamboo shoot, (**d**) carrot intake and (**e**) concentrated feed in different groups of giant pandas (dry matter). SGP: sub-adult giant panda, AGP: adult giant panda, GGP: geriatric giant panda. dark spots resprent the daily intake of individual giant panda in different groups. * *p* < 0.05, ** *p* < 0.01.

Fecal and diet samples were obtained from SGPs (*n* = 5), AGPs (*n* = 5) and GGPs (*n* = 11) of the China Conservation and Research Center for the Giant Panda (CCRCGP), Dujiangyan base. All of them were healthy with steady feed consumption. The diet composition (bamboo, bamboo shoots, concentrated feed, apples, and carrots) of these GPs were the same. Details are provided in Figure 1, which shows the feed composition and feed intake in different age groups of GPs. The amount of feed intake was calculated as the difference between feed offered and residual, which was recorded and weighed for each individual GP. Then the corresponding proportions of feed intake per day for each individual GP were collected and dried for nutrient concentration. Meanwhile, the excreted feces of each individual GP throughout the day were weighed and mixed, then about 5 kg were dried for nutrient digestibility analysis.

Fresh fecal samples were collected and sent to the laboratory within minutes. Under aseptic conditions, the exterior of the feces was stripped to avoid contamination, and the interior feces were taken for DNA extraction.

### 2.2. Chemical Analysis and Apparent Digestibility Calculation

All dried samples were ground and passed through a 0.25 mm sieve before analysis. According to the Association of Official Analytical Chemists (AOAC) analysis method, the acid-base lixiviation method was used to measure crude fiber (CF, method 962.09) [20], the Kjeldahl method was used to measure crude protein (CP, method 984.13), and ether extract (EE, method 920.39) was tested by the Soxhlet extraction method with diethyl ether [21]. An oven-drying method was adopted to determine the dry matter (DM, method 934.01) [21]. A total of 17 amino acids, including aspartic acid (Asp), threonine (Thr), serine (Ser), glutamic acid (Glu), glycine (Gly), alanine (Ala), valine (Val), isoleucine (Iso), leucine (Leu), tyrosine (Tyr), phenylalanine (Phe), lysine (Lys), histidine (His), arginine (Arg), proline (Pro), cysteine (Cys) and methionine (Met) were determined using the acid hydrolyzed method (GB/T 18246-2019 [22], China). Briefly, proteins in samples were hydrolyzed to amino acids under the action of 110 °C and 6 mol/L hydrochloric acid solution and separated by ion exchange chromatography and a ninhydrin column. After derivatization, proline was measured at 440 nm and other amino acids were measured at 570 nm. Apparent digestibility was calculated as follows: (%) = (amount of nutrient intake-amount of nutrient excreted in the feces)/amount of nutrient intake) × 100.

### 2.3. DNA Extraction and Amplicon Sequencing

Generally, each 50 g fecal sample was weighed and added to sterile phosphate buffered saline (PBS), and after oscillatory mixing the fecal suspension was centrifuged, and the centrifuged precipitate was collected for DNA extraction. Total genomic DNA was extracted using the DNeasy PowerSoil Kit (Qiagen, Hilden, Germany) according to the manufacturer’s instructions. Bacterial amplicon libraries were prepared by amplifying the 16 S rRNA V3-V4 region (341F: CCTAYGGGRBGCASCAG; 806R: GGACTACNNGGGTATCTAAT). Each PCR mixture consisted of 15 μL of Phusion^®^ High-Fidelity PCR Master Mix (New England Biolabs, Ipswich, MA, USA), 0.2 μM of primers, and 10 ng template DNA. Thermal cycling consisted of initial denaturation at 98 °C for 1 min, followed by 30 cycles of denaturation at 98 °C for 10 s, annealing at 50 °C for 30 s, and elongation at 72 °C for 30 s, with a final extension at 72 °C for 5 min. Then, the mixture of PCR products was purified with a Qiagen Gel Extraction Kit (Qiagen, Germany). The library was constructed using a TruSeq^®^ DNA PCR-Free Sample Preparation Kit and sequenced on an Illumina NovaSeq6000 platform, and the quality was assessed via the Qubit@ 2.0 Fluorometer (Thermo Scientific, Waltham, MA, USA) and Agilent Bioanalyzer 2100 system (Agilent, Santa Clara, CA, USA). Sequencing was conducted using the Illumina NovaSeq platform and 250 bp paired-end reads were generated (Illumina, San Diego, CA, USA).

### 2.4. Statistical Analysis

The clean tags were obtained after removing the barcode and primer sequence via FLASH (V1.2.7), http://ccb.jhu.edu/software/FLASH/ (accessed on 5 Marcch 2019) and quality filtering. Then, the chimera sequences were also filtered to obtain the effective tags by VSEARCH (2.14.1), https://github.com/torognes/vsearch/ (accessed on 8 March 2019). Based on the effective tags of all samples, Operational Taxonomic Units (OTUs) were picked via UPARSE (V7.0.1001) using a 97% similarity threshold [23]. A representative sequence for each OTU was annotated via SILVA 132, http://www.arb-silva.de/, (accessed on 15 March 2019) [24], using mothur methods (threshold values 0.8–1.0) [23]. Subsequently, alpha diversity was calculated with QIIME (V1.9.1). The principal coordinate analysis (PCoA) that uses the binary_jaccard matrix was performed using WGCNA, the stat and ggplot2 package in R software (V2.15.3, https://www.r-project.org/, accessed on 3 April 2023). The *t*-test was performed using the *t*-test function of R. To analyze functional enrichment of bacterial communities, the Tax4Fun (1.0) was then used to predict and evaluate the discrepancy of the KEGG pathway. One-way analysis of variance (ANOVA) was used to analyze the significant differences of feed intake, nutrient level of feed intake, and apparent nutrient digestibility in different age groups of GPs via SPSS 27 (Version 27.0, International Business Machines Corporation Inc., New York, NY, USA), respectively. Partial correlation was used to analyze correlation coefficients and significance between the feed intake and related nutrient level via SPSS 27. The Spearman’s correlation between the gut microbiota and nutrient digestibility parameters were determined using the ggcor package within R. A *p* value less than 0.05 was considered statistically significant.

## 3. Results

### 3.1. Composition and Nutrient Level of GPs’ Diet

The average feed intake (dry weight) in different age groups were calculated (Figure 1). As the main food of GPs, the average bamboo intake of SGPs (6694.04 g) was significantly higher than that in GGPs (1603.42 g) (*p* < 0.01), while the average intake of bamboo shoots increased, from 248.40 g in SGPs to 533.18 g in GGPs. The AGPs generally consume significantly more apple (24.89 g) when compared with GGPs (18.10 g) (*p* < 0.05). Moreover, both concentrated feed and carrot had similar intake in different age groups.

The nutrient level of dietary feed in different age groups were tested (Table 1). Generally, CF was the main component, followed by CP and EE. The concentration of Asp was highest in tested amino acids and Glu, Leu, Phe, and Pro also showed a high content. The mean concentrations of nutrients in different age groups were compared and the results indicated most of the nutrient content in their daily diet decreased as GPs aged. In detail, there were slightly differences between SGPs and AGPs (*p* > 0.05), while SGPs gained significantly more CF (*p* < 0.01), Cys (*p* < 0.01), Phe (*p* < 0.05), and Pro (*p* < 0.05) than GGPs. However, the content of Met was increased from SGPs to GGPs.

Significant correlations between feed intake and nutrient concentrations were confirmed (Figure S1). As major dietary elements, bamboo and bamboo shoot intakes were closely related to nutrient concentrations in GPs’ diet. Our results indicated all nutrient concentrations (except Met) were significantly and positively correlated with bamboo intake. With the exception of CF and Cys, bamboo shoot intake showed significant and positive correlation with all other nutrient concentrations (*p* < 0.01). The carrot intake showed significantly negative correlation with Met only (*p* < 0.01). However, apple and concentrated feed intake at lower levels of consumption did not significantly affect nutritional concentrations in GPs’ diet.

### 3.2. Apparent Nutrient Digestibility in GPs

The apparent nutrient digestibility for different nutrients in GPs’ diet were calculated and are shown in Table 2. The apparent digestibility of CF (7.82–12.41%) was lower compared to CP (69.58–71.52%) and EE (56.86–73.45%). The effect of age on the nutrient utilization efficiency was observed, which showed that GGPs had weaker CF and EE extraction capacity compared to SGPs and AGPs. However, the apparent digestibility of CP in AGPs and GGPs was slightly higher than that in SGPs. The digestibility of amino acids including Asp, Glu, Leu, Tyr, Phe, Lys, and Met exhibited over 70% apparent digestibility in different groups, while apparent digestibility of Thr were lower than 60% in different groups. There was a significant effect of age on Cys and Met digestibility, as apparent digestibility of Cys was higher in SGPs than in GGPs (*p* < 0.05). Conversely, apparent digestibility of Met was higher in GGPs than in SGPs (*p* < 0.05).

### 3.3. Comparisons of Microbiota Diversity in Different Age of GPs

**Figure 2:**
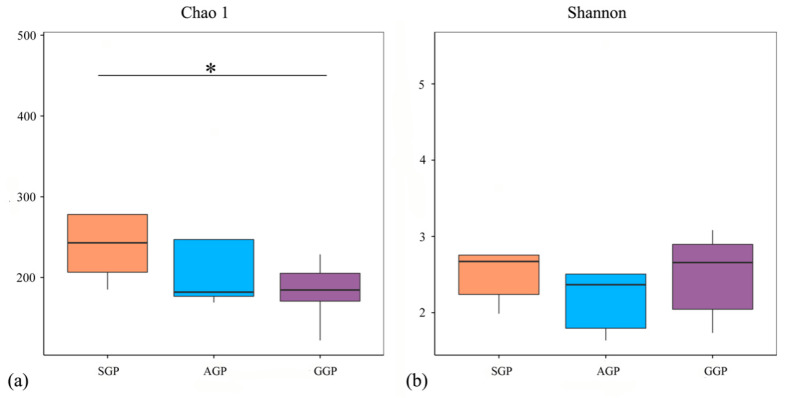
Alpha diversity of bacterial community in the gut of giant pandas of different age groups. (**a**) Chao 1, and (**b**) Shannon indices. SGP: sub-adult giant panda, AGP: adult giant panda, GGP: geriatric giant panda. * *p* < 0.05.

**Figure 3:**
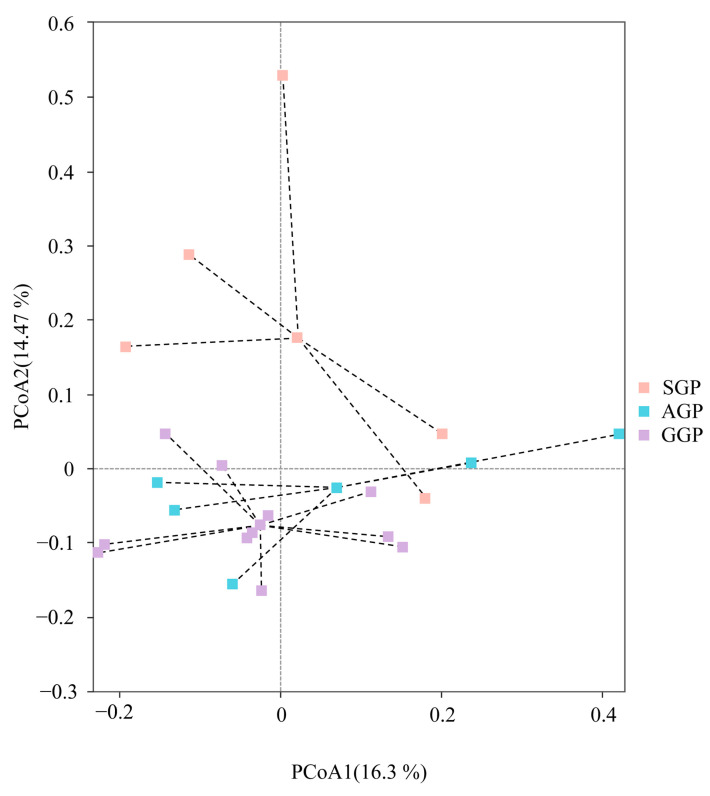
Principal Co-ordinates Analysis (PCoA) of bacterial community structure in the gut of giant pandas of different age groups. SGP: sub-adult giant panda, AGP: adult giant panda, GGP: geriatric giant panda.

In total, 1,339,230 effective sequences were obtained and yielded 631 OTUs. As shown in the Venn diagram (Figure S1), the number of OTUs per groups were identified (SGP, *n* = 428; AGP, *n* = 485; GGP, *n* = 409). Of these, 281 OTUs were shared by three groups. Meanwhile, the number of specific OTUs in SGP, AGP, and GGP was 66, 129, and 20, respectively. Alpha diversity indices, including Chao1 and Shannon indices were calculated for comparisons among the three groups (Figure 2 and Table S1). The Chao 1 index that reflected the bacterial richness was significantly lower in GGPs compared with SGPs (*p* < 0.05). However, the Shannon index indicated the bacterial diversity was approximately similar in different age groups of GPs. The comparison of beta diversity based on PCoA analysis is given in Figure 3. With increasing age, the SGPs had a trend of separation from AGPs and GGPs, while AGPs and GGPs cannot completely be distinguished.

### 3.4. Comparisons of Microbiota Community Composition in Different Age Groups of GPs

**Figure 4:**
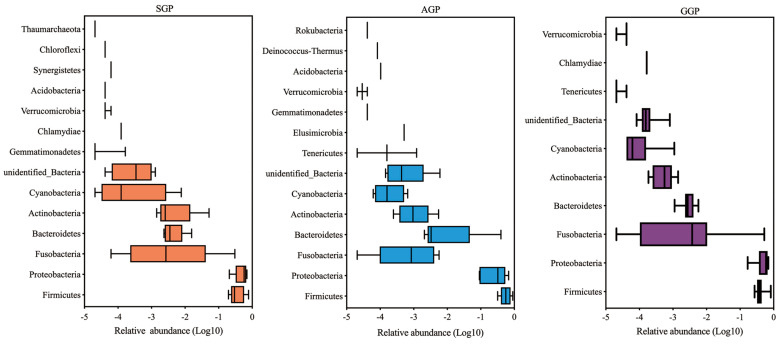
The composition of the bacterial community in different age groups of giant pandas at the phylum level. SGP: sub-adult giant panda, AGP: adult giant panda, GGP: geriatric giant panda.

**Figure 5:**
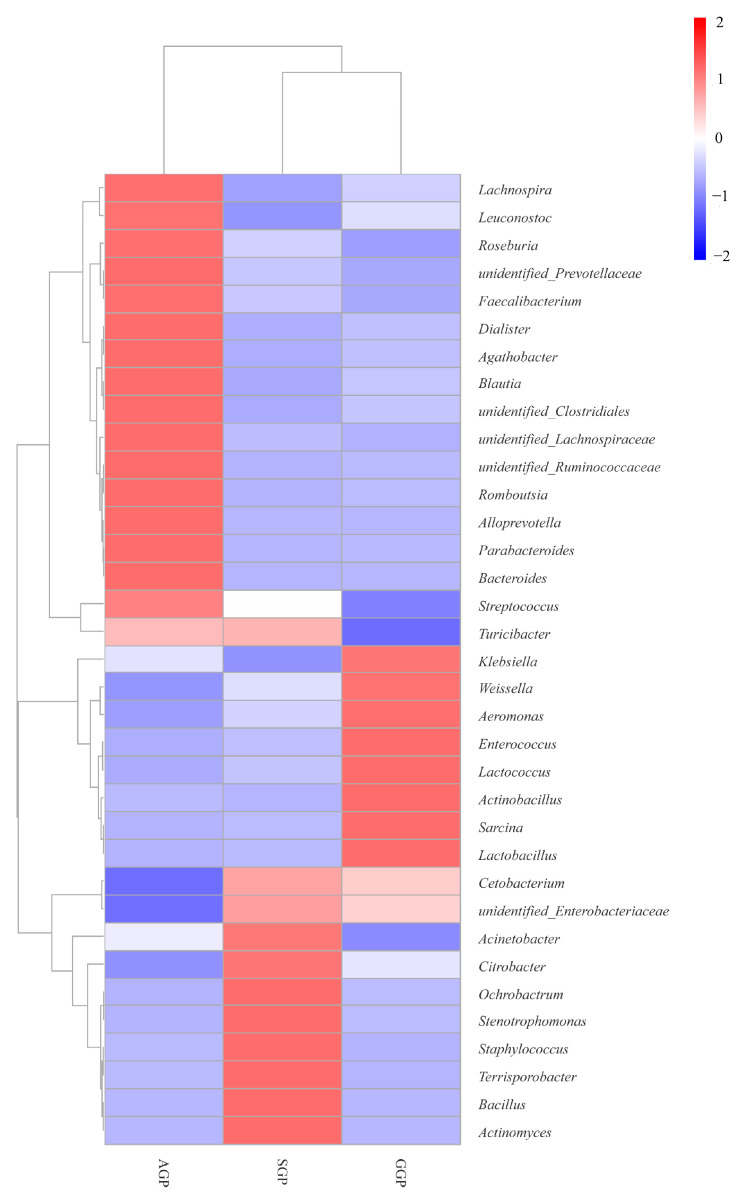
The relative abundance of dominant genera (top 35) in different age groups of giant pandas. SGP: sub-adult giant panda, AGP: adult giant panda, GGP: geriatric giant panda.

**Figure 6:**
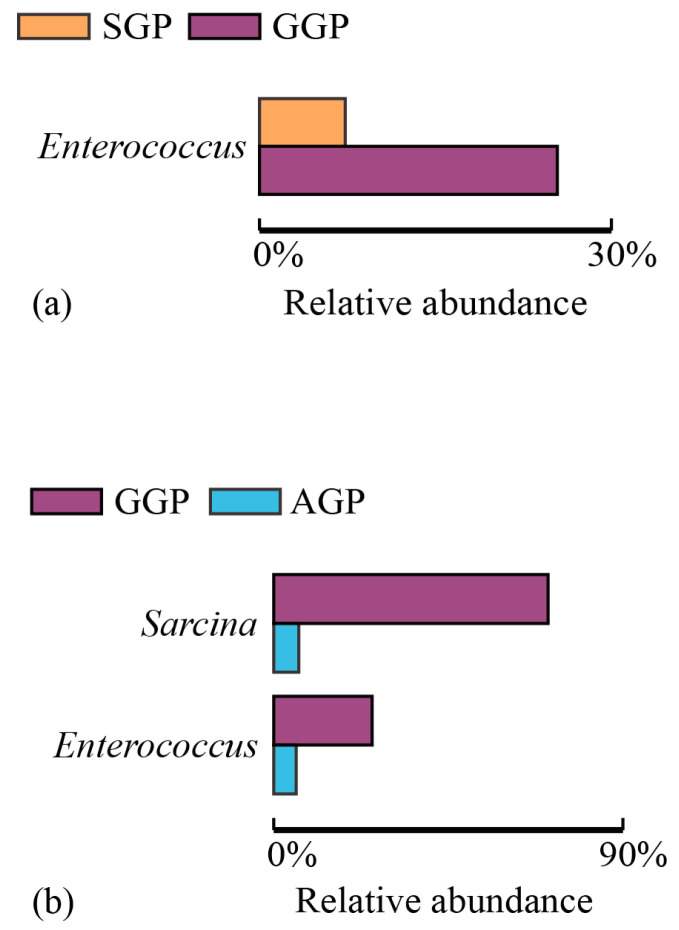
Significantly different bacterial taxa between (**a**) SGPs and GGPs, and (**b**) AGPs and GGPs identified by *t*-test. SGP: sub-adult giant panda, AGP: adult giant panda, GGP: geriatric giant panda.

In total, 18 phyla, 27 classes, 57 orders, 118 families, and 234 genera were obtained. There were 10 phyla and 169 genera found in GGPs, which showed lower diversity of community composition than that in in SGPs (14 phyla and 191 genera) and AGP (14 phyla and 181 genera). At the phylum level (Figure 4 and Table S2), there were 10 phyla found in GGPs, lower than that in SGPs (*n* = 14) and AGPs (*n* = 14). Similarly, seven phyla including Firmicutes, Proteobacteria, Bacteroidetes, Fusobacteria, Actinobacteria, Cyanobacteria, and unidentified_Bacteria were identified in all groups. In detail, Firmicutes and Proteobacteria were the most abundant phyla among different groups with a relative abundance of over 90%. In addition, the relative abundance of Actinobacteria decreased with age, dropping from 1.24% in AGPs to 0.06% in GGPs. At the genus level, the total number of genera in GGPs (*n* = 169) was lower than in SGPs (*n* = 191) and AGPs (*n* = 181). The 134 genera were shared among different age groups, and the top 35 genera in GPs were compared (Figure 5); the *unidentified_Enterobacteriaceae* and *Streptococcus* were the two most abundant genera. Notably, the relative abundance of these genera varied with different age groups. The relative abundance of *Enterococcus* was significantly higher in GGPs compared to the other groups, and the relative abundance of *Sarcina* in GGPs was significantly higher than in AGPs (*p* < 0.05) (Figure 6).

### 3.5. Comparisons of Microbiota Function in Different Age Groups of GPs

Tax4Fun was used to predict functional enrichment of bacterial communities. At KEGG pathway level 1 (Figure S2), metabolism (43.77–44.22%) was the primary KEGG pathway, followed by genetic information processing (24.56–25.02%), environmental information processing (14.36–15.28%), and cellular processes (6.01–6.12%). The relative abundance of each pathway in different groups was similar, except that a higher abundance of genetic information processing was observed in AGPs compared to SGPs and GGPs (*p* < 0.05). Furthermore, the enrichment of genes related to metabolism in the microbiota was analyzed at level 2, and the top 10 metabolism abundances are shown in Figure S3, in which carbohydrate metabolism (11.19–11.33%) had the highest abundance, followed by amino acid metabolism (8.01–8.02%), nucleotide metabolism (4.66–4.77%), and energy metabolism (3.81–3.89%); lipid metabolism has a relatively high abundance as well. The abundance did not differ significantly between groups. Among the top 10 pathways in metabolism, nucleotide metabolism was more enriched in AGPs compared to GGPs (*p* < 0.05), while the others did not differ significantly in different age groups.

### 3.6. Relationship between Gut Microbiota and Nutrient Apparent Digestibility in GPs

Significant correlations between apparent digestibility and alpha diversity (Shannon and Chao 1 indices) were investigated by the Spearman’s correlation analysis (Table S3) (*p* < 0.05 or 0.01). The apparent digestibility of the amino acids Cys, Pro, Phe, Tyr, Val, Ser, and Thr were significantly and positively correlated with bacterial richness (Chao 1 index), while no significant correlation was observed between bacterial diversity (Shannon index) and nutrient apparent digestibility.

**Figure 7:**
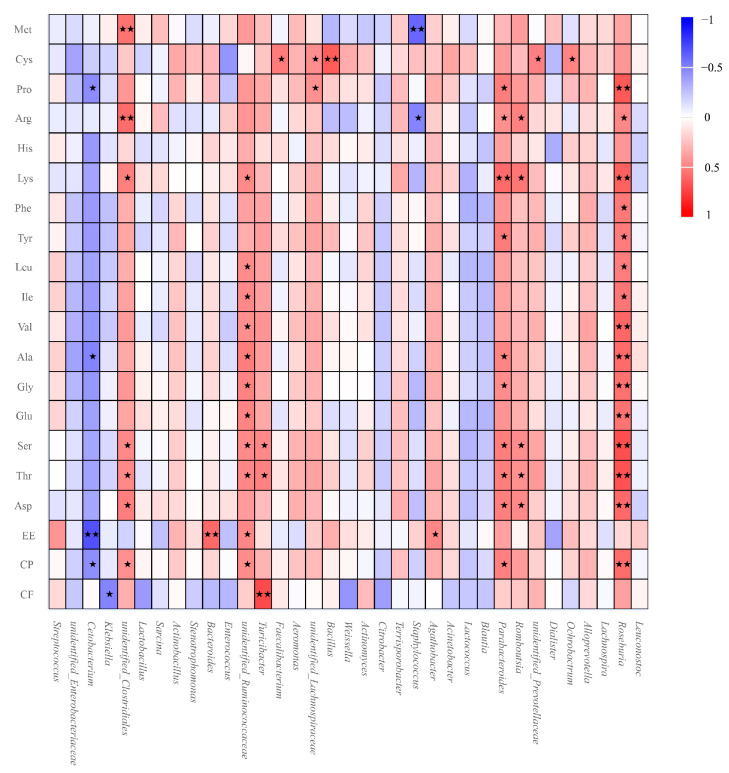
Spearman’s correlation analysis of relationship between bacterial abundance and apparent digestibility of dietary nutrients at genus level. Red color means positive correlation and blue color means negative correlation. CF, crude fiber; CP, crude protein; EE, ether extract; Asp, aspartic acid; Thr, threonine; Ser, serine; Glu, glutamic acid; Gly, glycine; Ala, alanine; Val, valine; Iso, isoleucine; Leu, leucine; Tyr, tyrosine; Phe, phenylalanine; Lys, lysine; His, histidine; Arg, arginine; Pro, proline; Cys, cysteine; Met, methionine. * *p* < 0.05 ** *p* < 0.01.

Correlations were assessed between the top 35 bacterial abundances at genus level and apparent digestibility of dietary nutrients (Figure 7). We found that some genera revealed a positive correlation with all nutrient apparent digestibility levels. Among these were, especially, *Roseburia*, *Romboutsia*, *Parabacteroides*, *Turicibacter*, *unidentified_Ruminococcaceae*, and *unidentified_Clostridiales*, which were significantly positively correlated with at least three nutrient apparent digestibility values (*p* < 0.05 or <0.01). In addition, all these genera showed a lower abundance in GGPs than in SGPs and AGPs. Notably, Ser and Thr could be significantly and positively affected by a number of bacterial genera, while CF only exhibited a positive correlation with the genus *Turicibacter* (*p* < 0.01). In contrast, three genera—including *Cetobacterium*, *Klebsiella*, and *Staphylococcus*—only revealed significantly negative correlation to nutrient apparent digestibility.

## 4. Discussion

As feed and management techniques have advanced, the average age of captive GPs has been rising in recent years. GPs’ diet composition and nutrients are receiving more attention, considering essential factors for maintaining the GPs’ health and lifespan [25]. It is generally accepted that feed intake changes as animals age and grow, while nutrient intake is affected by a combination of feed intake and dietary nutrient density [26,27]. Among five kinds of food intake, we found that nutrient intake was mainly significantly positively related to bamboo and bamboo shoot intake. Despite its reputation as an indigestible and low-nutritional-value feed [3,12], the large proportion of bamboo intake is the major approach to obtaining nutrients for GPs. The CF content in diet decreased significantly with the significant reduction in bamboo intake with aging, while there was no discernible variation in the amounts of CP, EE, or the majority of amino acids in GGPs when compared to the other groups. This is due to less fiber and more protein and fat in bamboo shoots compared with bamboo [28]. A previous study has suggested that bamboo shoots with protein levels ranging from 21.1–25.8 g/100 g on a dry weight basis are a potential source for proteins for human beings [29]. Similarly, the consumption of bamboo shoots also showed a strong favorable association with CP content in our investigation. Overall, these results imply that a suitable augmentation in the consumption of bamboo shoots can adequately counterbalance the nutritional deficiencies caused by the decrease in bamboo intake in GGP.

Based on results of apparent nutrient digestibility, the CF with the highest intake exhibited the lowest apparent digestibility among different age groups of GPs. We supposed that the abundant insoluble fiber in a bamboo diet, short digestive tracts, short food fermentation times, and the lack of genome encoding cellulose-digesting enzymes in GPs were the leading reasons [30,31,32]. Indeed, fragments of chewed bamboo leaves and bamboo stems are visible in the feces of GPs. In particular, this study found that CF digestibility in GGPs (7.82%) was significantly lower than in SGPs (12.41%). An earlier study reported by Yao et al. [3] has offered evidence that bamboo shoot-fed GGPs (7.78%) had lower CF digestibility than leaf-fed GGPs (22.01%). Hence, we proposed that a bamboo shoot-enriched diet in GGPs may result in decreased CF digestibility. Moreover, the functional decline of the gastrointestinal system and tooth wear could interfere with normal chewing and digestion of CF as age advances [9]. Nonetheless, the apparent digestibility of CP did not differ among different age groups, and was even slightly higher in the GGPs than in the SGPs. Higher CF consumption in AGPs may be related to the suppression of CP utilization, since Hu et al. [33] indicated that high amounts of CF in the diet have a negative impact on feed utilization, such as anti-nutrient factors and the decrease in nutrient digestibility. Moreover, a previous study showed that bamboo shoot consumption tends to stimulate the digestion of CP [28]. Combined with the mentioned findings, we suggest that bamboo shoots are a good source of CP and might be beneficial to the digestion of protein in GGPs.

There is growing recognition that besides their role as building blocks of proteins and polypeptides, amino acids are indispensable substrates for regulating key metabolic pathways involved in growth, reproduction, and immunity of the host [6]. With the current lack of knowledge regarding the intake and digestibility of amino acids in GPs, the results in this study could help regulate and improve the amino acid content in feed formulations of GPs. For GPs, both Asp and Glu content were higher in diets of different groups, and Asp plays a role in urea synthesis, gluconeogenesis, neurotransmission, and the regulation of hypothalamus function [34]. Glu is classified as a nonessential amino acid (NEEAs), but it becomes a conditionally essential amino acid (EAAs) under catabolic states since the body cannot synthesize sufficient amounts of glutamine, the monoamide of glutamic acid [35]. In the citric acid cycle, glutamine can be easily broken down and provide carbon for conversion into alpha-ketoglutarate, which serves as a vital energy source for rapidly dividing cells including epithelial and immune cells [36]. EAAs are key parameters in the diet, since they cannot be generated inside the body [37]. In the different age groups, among the EAAs the Leu intake was highest, which garnered considerable attention due to its anabolic effects on muscles and beneficial impact on glucose tolerance and lipid metabolism [38]. Considering the effect of age on the variation of amino acid intake and digestibility, we consequently found no significant difference in the digestibility of almost all amino acids among all the groups, indicating that aging had little effect on amino acid digestibility. However, most amino acid intake from dietary sources decreased with aging in GPs to different degrees. According to a previous study, elderly GPs are less responsive than younger individuals to anabolic stimulus with low doses of amino acid intake [39]. In other words, a higher level of amino acid consumption is required for elderly GPs to generate responses similar to those of younger individuals. Therefore, we further suggest that rational supplement of amino acids for GGPs to keep regular anabolism and maintain homeostasis.

It is acknowledged that the aging process, along with the ensuing changes in multiple physiological functions and feed intake, can directly affect the gut microbiota composition [40]. The findings showed that GGPs had a lower bacterial richness than AGPs and SGPs. Although no significant difference in bacterial diversity was observed, fewer bacterial phyla and genera were identified at phylum and genus level in the GGPs than in AGPs and SGPs. These results were in accordance with previous studies showing that aging reduces bacterial diversity in the GP’s gut, and they suggest it may be due to age-dependent differences in diet [19,41]. Moreover, dietary fibers can selectively enrich gut bacteria, and the short-chain fatty acids (SCFAs) produced by undigestible fiber degradation provide an energy source for gut microbes [42,43]. Thus, we hypothesized that decreased CF intake and digestibility with aging caused lower bacterial diversity and richness in GGPs. Firmicutes and Proteobacteria are the dominant phyla of bacteria in GPs and their abundance varied among different age groups; accumulated studies have confirmed this result [19,44]. Furthermore, there was a trend toward a decline in Actinobacteria abundance with age. Actinobacteria in the gastrointestinal tract of animals are a valuable source of enzymes that facilitate lignocellulose breakdown and enhance nutrient acquisition from a broad range of polysaccharides [45,46]. As a result, an increased Actinobacteria population in AGPs might offer more opportunities to improve bamboo fiber digestion. At the genus level, *Enterococcus* and *Sarcina* were significantly increased in GGPs. In the gut of GPs, *Enterococcus* is a lactobacillus with the ability to destroy plant cell walls and increase the content of soluble dietary fiber [33]. *Sarcina* has been linked to delaying gastric emptying, which increases carbohydrate substrates available to the host [47]. Thus, we hypothesized that increased *Sarcina* and *Enterococcus* abundance could promote the absorption of nutrients from high-fiber diets in GGPs.

Indeed, the nutrients derived from dietary intake modulates the gut microbiota of GPs, and in turn, changes in the gut microbiota could influence absorption of the micro and macronutrients in food [15,48]. Understanding the gut microbiota–nutrient interactions can offer valuable insights into potential therapeutic applications of food and dietary interventions, thus enabling personalized therapeutics and nutrition. In the present study, significant positive correlations were observed between bacterial richness and the apparent digestibility of several amino acids, such as Cys, Pro, Phe, Tyr, Val, Ser, and Thr, highlighting that the capacity to digest specific amino acids affects bacterial composition in the GP’s gut. Similarly, most studies have revealed that amino acids of dietary origin are the main constituents of intestinal bacterial protein in monogastric animals, which provide substances for nitrogen and carbon metabolism to produce bacterial cell components, supporting the growth and survival of bacteria [49,50,51]. In view of the fact that the ability of intestinal bacteria to utilize amino acids is species specific [52], we further investigated correlation between the abundance of dominant bacteria and nutrient apparent digestibility. Interestingly, all those genera exhibiting significant positive correlations with amino acids were at higher abundance in younger GPs, and significantly higher digestibility of Cys was found in SGPs compared to GGPs. Therefore, we suggested that amino acid digestibility reduction in GGPs was partly owed to the relatively lower abundance of bacteria that positively correlated with amino acids in GGPs. In addition, the higher abundance of the genus *Klebsiella* was negatively correlated with CF, which may be a result of lower CF digestibility in GGPs. These findings suggested to us the age-related changes in the gut microbiota have potential effects on the nutrient apparent digestibility.

## 5. Conclusions

In conclusion, our research demonstrated the variations of nutrient utilization and gut microbiota composition in different age groups of GPs. With aging, the dietary feed significantly changed, and less bamboo and more bamboo shoot consumption in GGPs significantly decreases the intake and apparent digestibility of CF, while the CP intake has no discernible variation. A significant difference in microbiota composition was noted in GGPs in comparison to SGPs and AGPs. The correlation between bacterial abundance and nutrient apparent digestibility highlighted that the age-related shift in gut microbiota has an influence on nutrient utilization. The results in this study can be used as a guide for nutritional management of GPs.

## Figures and Tables

**Table 1:** Nutrient level of basal diets in different age groups of GPs (dry matter).

Items	SGP	AGP	GGP
CF, g	1955.88 ± 647.72 ^a^	1102.39 ± 720.35	557.06 ± 633.48 ^b^
CP, g	332.1 ± 99.44	322.14 ± 111.75	264.85 ± 91.30
EE, g	69.73 ± 17.48	69.12 ± 18.89	56.94 ± 14.36
Thr, %	12.07 ± 3.65	11.28 ± 4.00	9.11 ± 3.25
Val, %	16.05 ± 4.91	14.66 ± 5.34	11.67 ± 4.37
Iso, %	11.72 ± 3.35	11.08 ± 3.62	8.97 ± 2.90
Leu, %	23.03 ± 6.31	21.49 ± 6.63	17.20 ± 5.29
Tyr, %	10.54 ± 3.60	9.16 ± 4.01	7.13 ± 3.31
Phe, %	16.78 ± 4.93 ^a^	14.62 ± 5.14	11.27 ± 4.20 ^b^
Lys, %	15.03 ± 4.75	14.80 ± 5.50	12.32 ± 4.53
His, %	7.08 ± 2.08	6.74 ± 2.29	5.50 ± 1.83
Met, %	9.82 ± 5.38	18.26 ± 9.86	19.28 ± 9.69
Asp, %	42.14 ± 13.64	41.88 ± 15.98	35.06 ± 13.31
Ser, %	15.71 ± 4.70	14.60 ± 5.12	11.73 ± 4.17
Glu, %	37.41 ± 9.53	37.08 ± 10.35	30.57 ± 7.91
Gly, %	12.41 ± 3.66	12.06 ± 4.10	9.94 ± 3.31
Ala, %	15.47 ± 4.58	14.81 ± 5.07	12.09 ± 4.11
Arg, %	11.75 ± 3.04	12.59 ± 3.66	10.82 ± 2.78
Pro, %	18.11 ± 5.43 ^a^	15.46 ± 5.66	11.78 ± 4.66 ^b^
Cys, %	10.06 ± 3.33 ^a^	6.10 ± 3.64	3.43 ± 3.14 ^b^

Note: All data are shown as mean ± standard deviation. Values followed by different letters (a and b) in each row are significantly different (*p* < 0.05). SGP: sub-adult giant panda, AGP: adult giant panda, GGP: geriatric giant panda; CF, crude fiber; CP, crude protein; EE, ether extract; Asp, aspartic acid; Thr, threonine; Ser, serine; Glu, glutamic acid; Gly, glycine; Ala, alanine; Val, valine; Iso, isoleucine; Leu, leucine; Tyr, tyrosine; Phe, phenylalanine; Lys, lysine; His, histidine; Arg, arginine; Pro, proline; Cys, cysteine; Met, methionine.

**Table 2:** The apparent digestibility (%) of dietary nutrients in different age groups of GPs.

Items	SGP	AGP	GGP
CF	12.41 ± 2.94 ^a^	11.77 ± 2.34	7.82 ± 4.07 ^b^
CP	69.58 ± 7.01	71.52 ± 7.44	70.70 ± 9.52
EE	63.96 ± 9.71	73.45 ± 10.17	56.86 ± 20.16
Thr	58.00 ± 8.10	57.38 ± 13.58	58.40 ± 11.96
Val	69.94 ± 7.42	70.02 ± 8.70	67.84 ± 9.71
Iso	69.52 ± 7.70	70.57 ± 7.40	68.18 ± 10.20
Leu	73.63 ± 7.26	74.60 ± 5.44	72.89 ± 9.80
Tyr	80.97 ± 4.38	78.57 ± 7.97	76.82 ± 7.14
Phe	77.47 ± 6.26	76.73 ± 4.97	74.43 ± 8.57
Lys	71.65 ± 7.83	75.14 ± 6.27	75.25 ± 9.21
His	68.52 ± 7.66	68.99 ± 9.04	69.23 ± 10.42
Met	81.36 ± 20.31 ^a^	93.98 ± 3.26	94.57 ± 4.33 ^b^
Asp	79.42 ± 4.73	80.65 ± 5.95	81.03 ± 6.85
Ser	71.05 ± 5.76	69.53 ± 9.19	70.47 ± 9.06
Glu	71.00 ± 6.89	72.46 ± 5.69	72.61 ± 8.16
Gly	61.04 ± 9.28	63.36 ± 8.70	61.77 ± 13.15
Ala	67.46 ± 8.61	69.98 ± 6.38	66.70 ± 11.82
Arg	66.54 ± 8.09	72.28 ± 6.35	74.68 ± 11.26
Pro	73.16 ± 7.13	72.12 ± 8.14	69.52 ± 8.67
Cys	83.70 ± 8.43 ^a^	67.93 ± 25.64	56.19 ± 21.57 ^b^

Note: All data are shown as mean ± standard deviation. Values followed by different letters (a and b) in each row are significantly different (*p* < 0.05). CF, crude fiber; CP, crude protein; EE, ether extract; Asp, aspartic acid; Thr, threonine; Ser, serine; Glu, glutamic acid; Gly, glycine; Ala, alanine; Val, valine; Iso, isoleucine; Leu, leucine; Tyr, tyrosine; Phe, phenylalanine; Lys, lysine; His, histidine; Arg, arginine; Pro, proline; Cys, cysteine; Met, methionine.

## Data Availability

Raw data for microbiota sequencing are available from ncbi, bioproject PRJNA1130398. All other data involved in this study have been published in manuscripts and Supplementary Materials.

## Supplementary Materials

The following supporting information can be downloaded at: https://www.mdpi.com/article/10.3390/ani14162324/s1, Figure S1: Venn diagram showing the unique and shared OTUs among different age groups of giant pandas; Figure S2: The correlations among different feed intake and nutrient concentration; Figure S3: Prediction of enrichment bacteria function in KEGG pathways level 1 via Tax4Fun; Figure S4: Prediction of enrichment of bacterial function in KEGG pathways level 2 via Tax4Fun. Table S1: Spearman’s correlation coefficients between apparent digestibility and alpha diversities; Table S2: The relative abundance of bacterial community of giant pandas at the phylum level; Table S3: Spearman’s correlation coefficients between apparent digestibility and alpha diversities.

## Abbreviations

GP: giant panda; SGP: sub-adult giant panda; AGP: adult giant panda; GGP: geriatric giant panda; CF: crude fiber; CP: crude protein; EE: ether extract; Asp: aspartic acid; Thr: threonine; Ser: serine; Glu: glutamic acid; Gly: glycine; Ala: alanine; Val: valine; Iso: isoleucine; Leu: leucine; Tyr: tyrosine; Phe: phenylalanine; Lys: lysine; His: histidine; Arg: arginine; Pro: proline; Cys: cysteine; Met: methionine; NEEA: nonessential amino acid; EEA: essential amino acid; SCFA: short-chain fatty acid.
