# Supplementary material for: Nutrient Utilization and Gut Microbiota Composition in Giant Pandas of Different Age Groups
Source: Animals (Basel). 2024 Aug 12;14(16):2324. doi: 10.3390/ani14162324 (PMC11350801; doi:10.3390/ani14162324)
Supplement: Supplementary file 1 [file animals-14-02324-s001.zip › animals-3108887-supplementary.pdf]

Supplemental material

Legends for Supplemental Material

Figure S1 Venn diagram showing the unique and shared OTUs among different age groups of giant pandas. SGP: sub-adult giant panda; AGP: adult giant panda; GGP: geriatric giant panda

Figure S2 The correlations among different feed intake and nutrient concentration. ** P<0.01

Figure S3 Prediction of enrichment bacteria function in KEGG pathways level1 via Tax4Fun. SGP: sub-adult giant panda; AGP: adult giant panda; GGP: geriatric giant panda

Figure S4 Prediction of enrichment bacteria function in KEGG pathways level 2 via Tax4Fun. SGP: sub-adult giant panda; AGP: adult giant panda; GGP: geriatric giant panda

Table S1 Spearman's correlation coefficients between apparent digestibility and alpha diversities. CF, crude fiber; CP, crude protein; EE, ether extract; Asp, aspartic acid; Thr, threonine; Ser, serine; Glu, glutamic acid; Gly, glycine; Ala, alanine; Val, valine; Iso, isoleucine; Leu, leucine; Tyr, tyrosine; Phe, phenylalanine; Lys, lysine; His, histidine; Arg, arginine; Pro, proline; Cys, cysteine; Met, methionine. *: $p<0.05$; **: $p<0.01$

Table S2 The relative abundance of bacteria community of giant pandas at the phylum level. SGP: sub-adult giant panda, AGP: adult giant panda, GGP: geriatric giant panda.

Table S3 Spearman's correlation coefficients between apparent digestibility and alpha diversities. CF, crude fiber; CP, crude protein; EE, ether extract; Asp, aspartic acid; Thr, threonine; Ser, serine; Glu, glutamic acid; Gly, glycine; Ala, alanine; Val, valine; Iso, isoleucine; Leu, leucine; Tyr, tyrosine; Phe, phenylalanine; Lys, lysine; His, histidine; Arg, arginine; Pro, proline; Cys, cysteine; Met, methionine. *: $p<0.05$; **: $p<0.01$

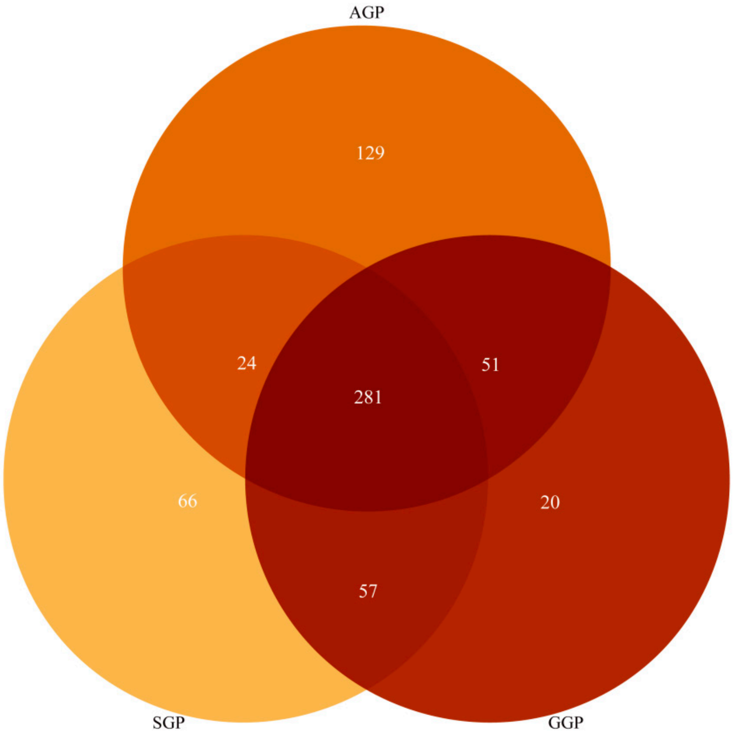

Figure S1 Venn diagram showing the unique and shared OTUs among different age groups of giant pandas. SGP: sub-adult giant panda; AGP: adult giant panda; GGP: geriatric giant panda

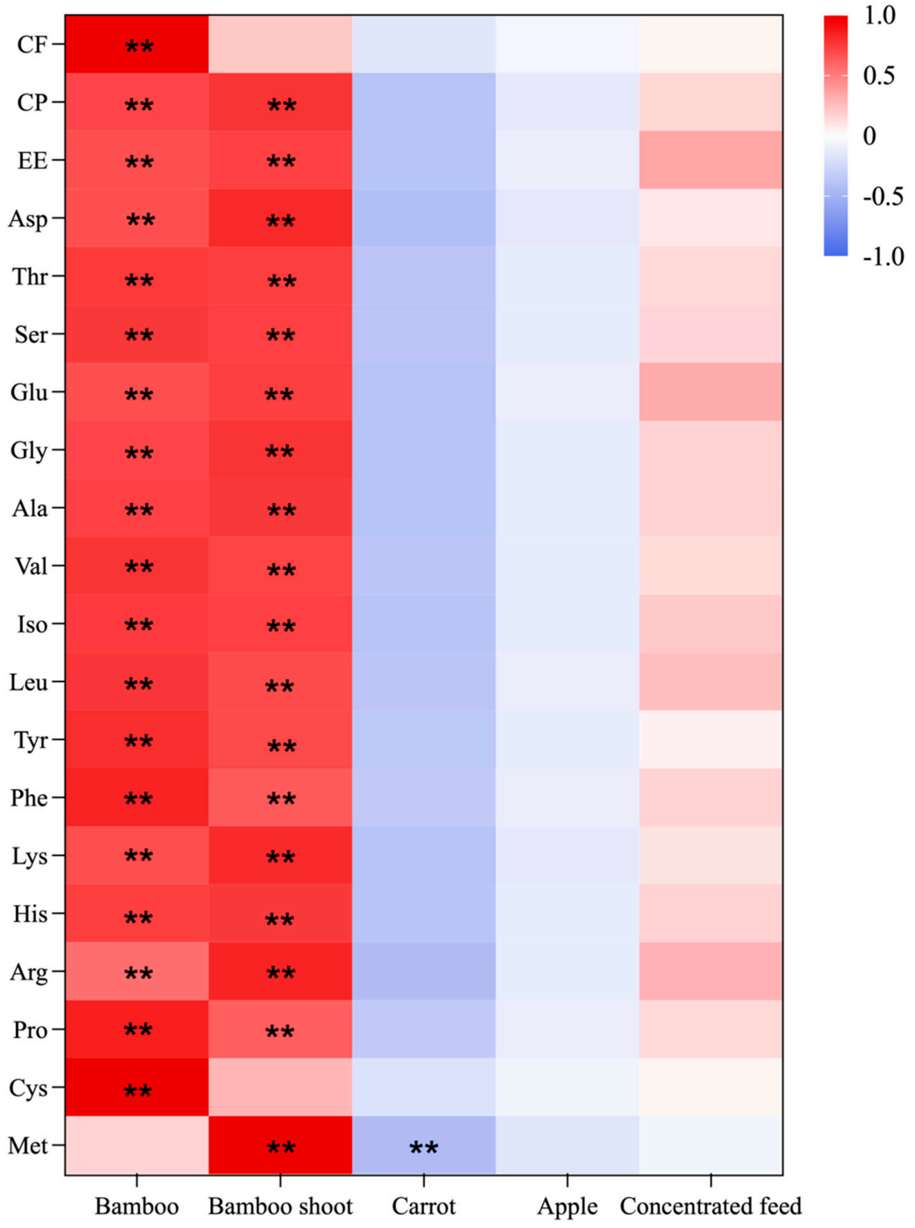

Figure S2 The correlations among different feed intake and nutrient concentration.
** P<0.01

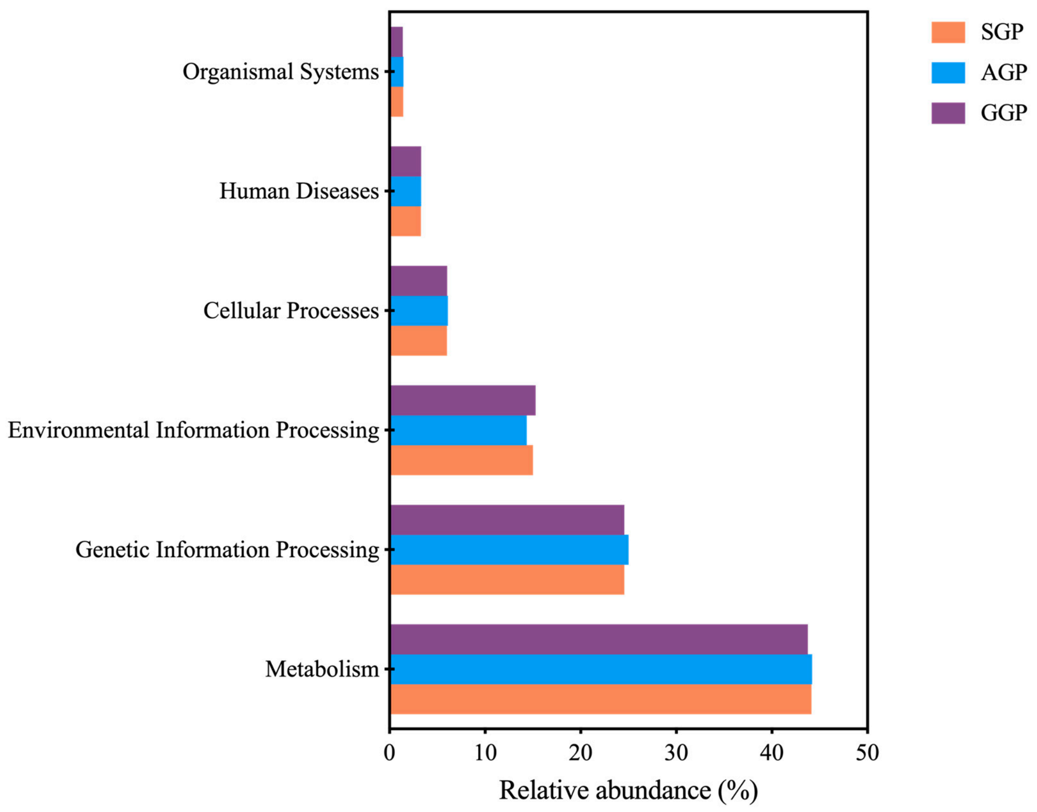

Figure S3 Prediction of enrichment bacteria function in KEGG pathways level1 via Tax4Fun. SGP: sub-adult giant panda; AGP: adult giant panda; GGP: geriatric giant panda

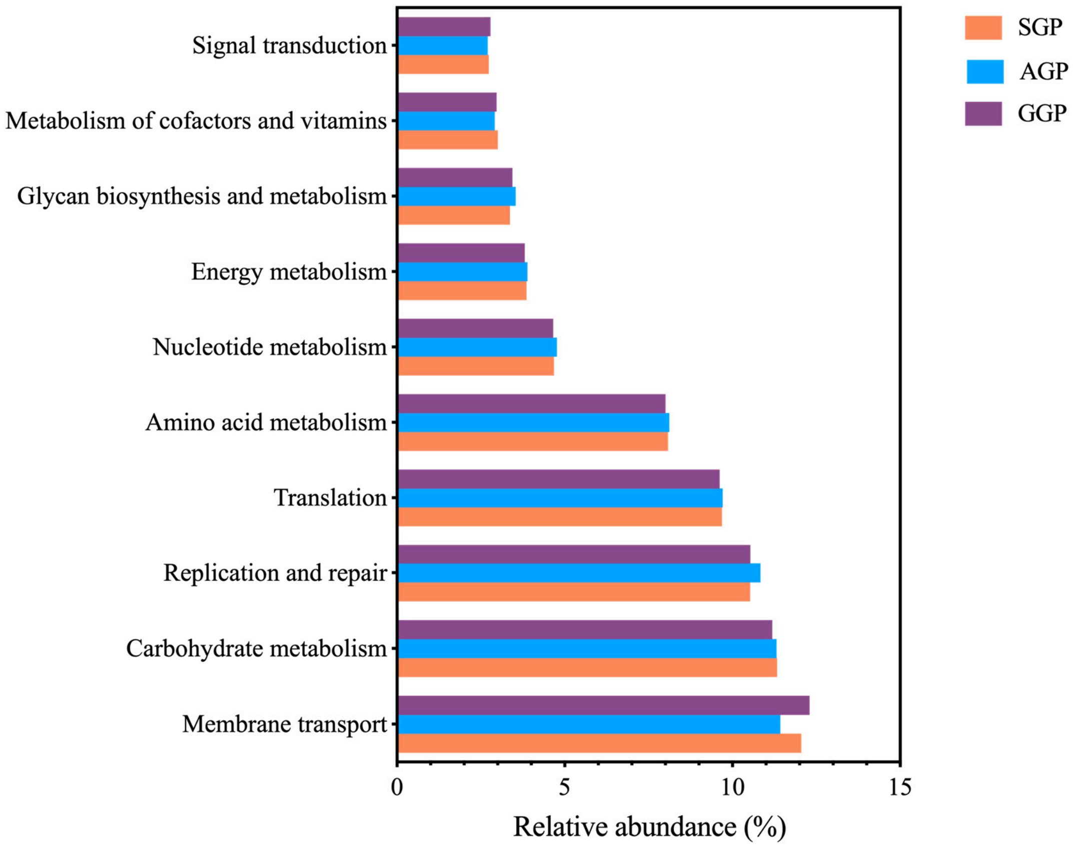

Figure S4 Prediction of enrichment bacteria function in KEGG pathways level 2 via Tax4Fun. SGP: sub-adult giant panda; AGP: adult giant panda; GGP: geriatric giant panda

Table S1 Spearman's correlation coefficients between apparent digestibility and alpha diversities

Items	Shannon	Chao1
CF	-0.09	0.32
CP	0.07	0.35
EE	-0.20	0.41
Asp	0.15	0.36
Thr	0.11	0.54*
Ser	0.08	0.52*
Glu	-0.04	0.31
Gly	0.02	0.32
Ala	0.08	0.36
Val	0.11	0.47*
Iso	0.01	0.39
Leu	0.00	0.41
Tyr	0.09	0.58**
Phe	0.08	0.51*
Lys	0.12	0.37
His	0.00	0.39
Arg	0.00	0.13
Pro	0.06	0.50*
Cys	0.36	0.57**
Met	-0.01	-0.10

CF, crude fiber; CP, crude protein; EE, ether extract; Asp, aspartic acid; Thr, threonine; Ser, serine; Glu, glutamic acid; Gly, glycine; Ala, alanine; Val, valine; Iso, isoleucine; Leu, leucine; Tyr, tyrosine; Phe, phenylalanine; Lys, lysine; His, histidine; Arg, arginine; Pro, proline; Cys, cysteine; Met, methionine.*: p<0.05; **: p<0.01

Table S2 The relative abundance of bacteria community of giant pandas at the phylum level

Phyla	SGP	AGP	GGP
Firmicutes	39.5629%	58.8687%	42.2218%
Proteobacteria	52.1223%	32.2886%	52.0987%
Fusobacteria	6.2809%	0.1981%	5.2642%
Bacteroidetes	0.5712%	8.2603%	0.3133%
Actinobacteria	1.2390%	0.1725%	0.0630%
Cyanobacteria	0.1589%	0.0215%	0.0126%
unidentified_Bacteria	0.0413%	0.1486%	0.0195%
Tenericutes	0.0000%	0.0248%	0.0008%
Elusimicrobia	0.0000%	0.0103%	0.0000%
Gemmatimonadetes	0.0041%	0.0008%	0.0000%
Chlamydiae	0.0025%	0.0000%	0.0015%
Verrucomicrobia	0.0054%	0.0012%	0.0015%
Acidobacteria	0.0008%	0.0021%	0.0000%
Deinococcus-Thermus	0.0000%	0.0017%	0.0000%
Synergistetes	0.0012%	0.0000%	0.0000%
Rokubacteria	0.0000%	0.0008%	0.0000%
Chloroflexi	0.0008%	0.0000%	0.0000%
Thaumarchaeota	0.0004%	0.0000%	0.0000%

SGP: sub-adult giant panda, AGP: adult giant panda, GGP: geriatric giant panda.

Table S3 Spearman's correlation coefficients between apparent digestibility and alpha diversities

Items	Chao1	Shannon
CF	0.32	-0.09
CP	0.35	0.07
EE	0.41	-0.20
Asp	0.36	0.15
Thr	0.54*	0.11
Ser	0.52*	0.08
Glu	0.31	-0.04
Gly	0.32	0.02
Ala	0.36	0.08
Val	0.47*	0.11
Iso	0.39	0.01
Leu	0.41	0.00
Tyr	0.58**	0.09
Phe	0.51*	0.08
Lys	0.37	0.12
His	0.39	0.00
Arg	0.13	0.00
Pro	0.50*	0.06
Cys	0.57**	0.36
Met	-0.10	-0.01

CF, crude fiber; CP, crude protein; EE, ether extract; Asp, aspartic acid; Thr, threonine; Ser, serine; Glu, glutamic acid; Gly, glycine; Ala, alanine; Val, valine; Iso, isoleucine; Leu, leucine; Tyr, tyrosine; Phe, phenylalanine; Lys, lysine; His, histidine; Arg, arginine; Pro, proline; Cys, cysteine; Met, methionine. *: $p<0.05$; **: $p<0.01$
